# Quality Assessment of Studies Included in Cochrane Oral Health Systematic Reviews: A Meta-Research

**DOI:** 10.3390/ijerph18147284

**Published:** 2021-07-07

**Authors:** Ahmad Sofi-Mahmudi, Pouria Iranparvar, Maryam Shakiba, Erfan Shamsoddin, Hossein Mohammad-Rahimi, Sadaf Naseri, Parisa Motie, Marcos Roberto Tovani-Palone, Bita Mesgarpour

**Affiliations:** 1 Cochrane Iran Associate Centre, National Institute for Medical Research Development (NIMAD), Tehran 1419693111, Iran; sofimahmudi@research.ac.ir (A.S.-M.); pouria.ipv@gmail.com (P.I.); Maryam.shakiba1375@gmail.com (M.S.); shamsoddin@research.ac.ir (E.S.); 2 Dental Research Center, Research Institute of Dental Sciences, Shahid Beheshti University of Medical Sciences, Tehran 1983969411, Iran; ramtin.rhm@gmail.com; 3 School of Dentistry, Shahid Beheshti University of Medical Sciences, Tehran 1983969411, Iran; sadafnasseri7701@gmail.com (S.N.); pm.motie@gmail.com (P.M.); 4 Ribeirão Preto Medical School, University of São Paulo, Ribeirão Preto 14049-900, Brazil

**Keywords:** bias, clinical trial, systematic review, dentistry, evidence-based dentistry, risk

## Abstract

Objectives: To assess the Risk of Bias (RoB) and other characteristics of published randomised clinical trials within Cochrane oral health systematic reviews. Materials and methods: All the published clinical trials within Cochrane oral health systematic reviews until 1 June 2020 were identified and examined. RoB was assessed for all the included clinical trials according to the Cochrane review standards. The Overall Risk of Bias (ORoB) was defined in this study using Cochrane’s RoB tool-v2. Descriptive analyses were carried out to determine the frequency of each variable in the study sample. Results: Out of a total of 2565 included studies, the majority (*n* = 1600) had sample sizes of 50 or higher. Regarding blinding, 907 studies were labelled as double-blind. Among the various domains of bias, the performance bias showed the highest rate of high risk (31.4%). Almost half of the studies had a high ORoB, compared to 11.1% with a low ORoB. The studies that used placebos had a higher percentage of low ORoB (14.8% vs. 10.7%). Additionally, the double- and triple-blind studies had higher percentages of low ORoB (23.6% and 23.3%, respectively), while the studies with a crossover design had the highest percentage of low ORoB (28.8%). Conclusion: The RoB of oral health studies published as Cochrane reviews was deemed high.

## 1. Introduction

The quality assessment of studies should always consider both internal and external validities [1], which are critical aspects of any scientific project; notwithstanding, internal validity is more relevant to empirical studies [2]. The Risk of Bias (RoB) is a good measure of the internal validity of a study [3,4]. Bias in clinical studies leads to the over- or under-estimation of treatment outcomes [5]. From a clinical perspective, bias can lead to the application of an intervention that may not be effective or that may even be potentially harmful [3]. Although randomised controlled trials (RCTs) are widely considered the gold standard for therapeutic clinical research in order to measure the effectiveness of new medical interventions, they are also prone to bias due to flaws in their design, conduct, analysis or reporting [6].

The Cochrane Database of Systematic Reviews is proposed as the largest and most recognised scientific database of systematic reviews and meta-analyses in health sciences that publishes high-quality systematic reviews with specific methodological protocols [7]. In the case of RoB, Cochrane reviews use a specific domain-based assessment tool; this tool has been changed over the years, with the latest edition introduced in 2008 [8]. Currently, its domains consist of ‘sequence generation’, ‘allocation concealment’, ‘blinding of participants and personnel’, ‘other potential threats to validity (other sources of bias)’, ‘blinding of outcome assessment (blinding of the examiner)’, ‘incomplete outcome data’, and ‘selective outcome reporting’. The Cochrane assessment tool for RoB is a scale with three levels, namely, ‘Low-risk’, ‘High-risk’ and ‘Unclear-risk’. The first two levels are self-explanatory, and the unclear-risk choice is assigned when there is not enough evidence available (reported by the study) to explicitly draw a conclusion about any specific domain. The primary sources of support and reliance for Cochrane judgments are pieces of evidence or citations from the assessed paper, or any rational inferences and reviews drawn from the author’s explanations [9].

Despite all the valuable efforts made in the past few years, there is still a considerable gap between the available evidence and the common clinical procedures in dentistry. As a result, in many cases, it may be difficult for practitioners to extract the needed data from relevant studies. Moreover, there are also studies with inadequate allocation concealment and a high RoB, which tend to change empirical results in a clinical setting [10]. Thus, without a way to provide dentists with high-quality evidence to reinforce their decision-making process, dentists will face substantial stress, and their decision-making will take longer in clinical settings. Therefore, the present study aimed to assess the RoB and its trend and other characteristics of RCTs, as well as RoB in defined groups of RCTs within Cochrane oral health systematic reviews on the subject of dentistry and oral health. This will help the oral health research community better understand the current status of existing evidence and may be a first step towards reducing research waste and providing higher quality evidence in dentistry. Further suggestions to be considered by future Cochrane review teams will be presented toward the end of this article.

## 2. Materials and Methods

This study is a secondary analysis of clinical trials within Cochrane oral health systematic reviews. All the judgements concerning the quality of the RCTs were made by Cochrane review authors.

### 2.1. Search Strategy

The “dentistry and oral health” filter was used to search in the Cochrane Library’s “Search Reviews” section on 1 June 2020. All the reviews in this category were included in the data extraction step.

### 2.2. Identification and Selection of Primary Studies

**Figure 1:**
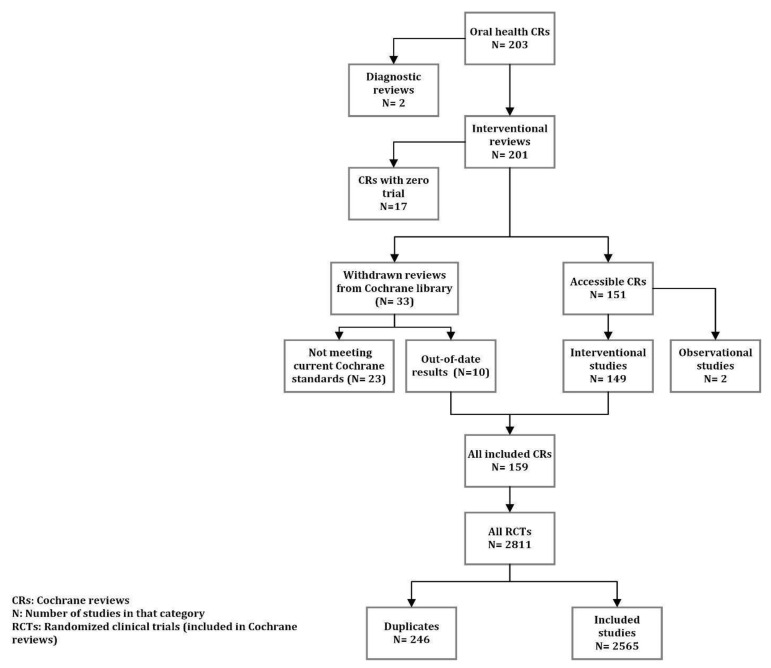
Flowchart for study selection strategy.

Primary studies were identified using the references from Cochrane reviews, and their data were extracted based on the details in tables of the included studies. The diagnostic reviews and systematic reviews on observational studies were excluded, given that they have different RoB tools to assess the studies included in them, which makes it impossible to compare them with systematic reviews of interventional studies. Figure 1 shows the detailed flow diagram of steps taken to select the eligible studies.

### 2.3. Data Extraction and Management

This step of the study was carried out involving all the included trials by seven independent authors (A.S.-M., P.I., M.S., E.S., H.M., S.N., and P.M.), and the results were entered directly into preformatted Excel 2016 spreadsheets. These spreadsheets were then merged into one file and rechecked to avoid any errors and inconsistencies among the data extractors (A.S.-M., P.I., and M.S.). Data to be extracted were divided into four categories: (1) general information of Cochrane reviews, including the type of review, the subject, the number of included trials; (2) basic information on the RCTs, e.g., title, first author, year of publication, Journal; (3) the information reported in the RCTs (the “Characteristics of the included studies” section), which basically characterised the main methodological features of each RCT; (4) the RoB assessment of the RCTs.

Given that the blinding process was assessed differently by the Cochrane review authors, the RoB assessments were performed in different ways. In some studies, blinding involved two subgroups, i.e., blinding of participants and personnel (performance bias) and blinding of outcome assessment (detection bias); however, in other studies, blinding was taken as one item. In rare instances, blinding involved two categories, including subjective and objective outcomes or a range; namely, assessor, analyst, participants, and caregivers. In these cases, an aggregated form was provided; if one or more subcategories were reported as high-risk, the whole category of blinding bias was considered high-risk, and if all were low risk, the whole category was considered low-risk. Otherwise, the blinding bias was reported as unclear.

Some other RoB assessment items were rarely reported; for instance, funding bias, intention to treat bias, sample size bias, for-profit bias, and power calculation bias. Due to the low frequency of these items, these kinds of biases were not extracted in this study. In addition, a new variable was defined to aggregate the full results of the assessments, named “Overall Risk of Bias” (ORoB). The criteria for determining the ORoB rating were the same as for the “Overall Bias” assessment, using Cochrane’s RoB tool -version 2 in both cases [11]. If any of the bias domains were considered high-risk, the ORoB rating was considered high. In the absence of high-risk biases, unclear risk assessment biases were perceived in the same way; otherwise, the ORoB rating was considered low. RCTs that appeared in more than one Cochrane review were excluded if their RoB assessments were not consistent across the five domains that were assessed. Assessing RoB is subjective; therefore, sometimes the assessments of different authors do not match.

When including RCTs in this study, several groups were considered, namely, quasi-experimental (controlled before-after, interrupted time series, and non-randomised designs), parallel RCT, crossover RCT, cluster RCT, split-mouth RCT, and repeated-measures study designs. Finally, to examine the Cochrane review authors’ subjectivity and bias of judgement, the studies were distinguished by RoB domains. Cochrane reviews that included the same studies, either with the same or with different RoB assessments, were listed and grouped into different categories based on the included titles.

### 2.4. Statistical Analysis

The extracted data were analysed using R v3.6.0 (26 April 2019) (R Core Team, R Foundation for Statistical Computing, Vienna, Austria. http://www.R-Project.org accessed on 15 May 2019). Mean and standard deviations were reported for the continuous quantitative variables and frequencies for the qualitative data.

## 3. Results

### 3.1. Characteristics of Cochrane Reviews

Of the 8293 Cochrane reviews found, 203 (2.4%) were on dentistry and oral health (23rd rank among the subjects in terms of the number of reviews). The majority of reviews were interventional (99.0%) and there were two diagnostic studies. The review subjects were mainly concerned with dental caries (24.6%), craniofacial anomalies (18.7%), and oral and maxillofacial surgery (15.8%). Seventeen reviews (8.4%) included zero studies, and 33 (16.3%) reviews were withdrawn for different reasons, mainly because they were out-of-date (75.8%) and did not meet the current Cochrane methodological standards (54.5%). Nonetheless, ten of the studies withdrawn by Cochrane were included, as they were only out-of-date and were still methodologically satisfactory based on the latest Cochrane standards. Additionally, two reviews included only observational studies and we excluded them. Hence, 159 interventional reviews (80.3%) provided the data needed for our research.

The mean and median of the number of the studies included in these reviews were 18.7 (SD = 26.0) and 10 (range: 1–154), respectively. Four reviews (4/203, 2.0%) had more than 100 included studies and 80 (80/203, 39.4%) had fewer than ten (excluding the zero studies). The subjects of the three most-cited reviews were oral cancer and precancerous lesions (N = 309), oral and maxillofacial surgery (N = 305), and dental caries (N = 275) according to the Web of Science. Nonetheless, reviews of antibiotic therapy (78.5, N = 2), oral lichen planus (73.5, N = 2), and periodontal diseases (69.6, N = 17) had the highest mean number of citations.

### 3.2. Characteristics of the Included Studies

**Figure 2:**
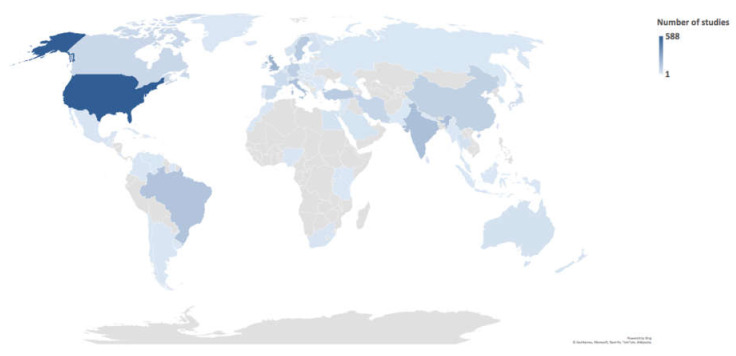
World map for the distribution of conducting sites.

The reviews included 2811 studies, among which 246 were duplicated. Therefore, a total of 2565 studies were included in our analysis. Almost two-thirds of these studies (*n* = 1666) were published after the year 2000. The distribution of locations where the studies were conducted was heterogeneous. The USA, the UK, and India had the highest number of studies with 588 (22.9%), 221 (8.6%), and 164 (6.4%) studies, respectively. Sixty-eight studies were conducted through international collaborations. Figure 2 shows the distribution of the location of the studies.

Out of the 614 study sources, four were unpublished and one was a dissertation from the University of Sao Paulo, Brazil. However, 117 journals had more than three studies included in the Cochrane reviews. Most of these journals were published in the USA (47.9%) and the UK (26.4%). According to the SCImago Journal Rank (SJR) quartiles (2019) (https://www.scimagojr.com/journalrank.php accessed on 15 May 2019), over half of these journals were categorised as Q1 (52.1%), followed by Q2 (25.6%). The most frequently covered subjects in the journals were dentistry (miscellaneous; 41.0%), followed by medicine (miscellaneous; 10.3%) and oral surgery (10.3%). The following journals represented the largest inclusions in the Cochrane reviews: *Journal of Clinical Periodontology* (*n* = 108, 4.2%), *Journal of Periodontology* (*n* = 103, 4.0%), and *Journal of the American Dental Association* (*n* = 67, 2.6%).

Most of the studies (*n* = 1600, 62.4%) had sample sizes of 50 or more. The mean and median of the sample sizes were 1017 (SD = 3875.1) and 303 (range: 3–191,873), respectively, and 86 studies had sample sizes greater than 1000. In contrast, 4.3% of the studies had fewer than 20 participants. The sample size was not reported in the tables in 17 studies. More than three-fourths of the included studies were parallel RCTs (*n* = 2040), followed by split-mouth (*n* = 253), crossover (*n* = 154), and quasi-experimental (*n* = 72) studies. The design was unclear in one study. From a methodological point of view, 787 (30.7%) studies reported having a placebo arm as the control group. As for blinding, 907 (35.3%) studies were labelled as double-blinded, 60 studies (2.4%) as triple-blinded, while 425 (16.6%) studies did not use any blinding in their design. The number of double- and triple-blinded studies was 967, and 791 of them (81.8%) had a low risk of performance bias.

**Figure 3:**
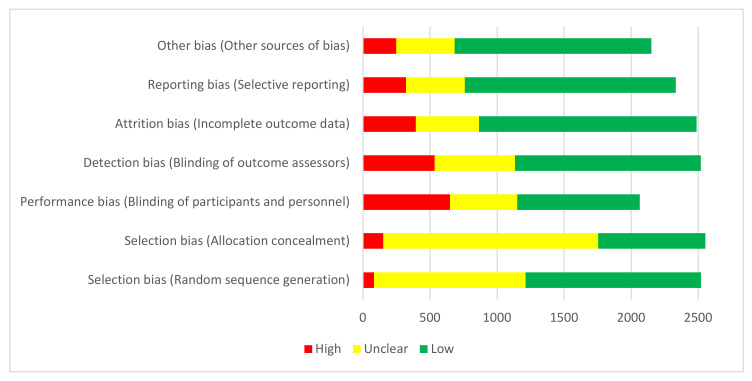
Risk of Bias assessment for all domains of RCTs included and assessed in Cochrane Oral Health systematic reviews.

**Figure 4:**
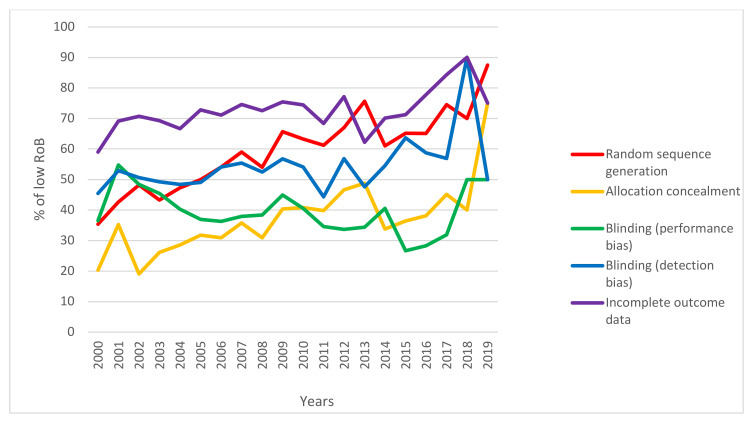
The trend of low RoB (Risk of Bias) for five domains of Cochrane tool in oral health RCTs during 2000–2019.

Almost all domains of the RoB tool were assessed in all the reviews. However, in 45 (1.8%) and 11 (0.4%) of the interventional studies, respectively, random sequence generation and allocation concealment were not assessed by the reviewers. Among the various domains of bias, the performance bias (blinding of the participants and personnel) showed the highest rate of high risk (31.4%). In contrast, other sources of bias had the highest rate of low risk (68.2%). Figure 3 presents the overall assessment of each domain of the RoB tool. According to Figure 4, the random sequence generation data have continuously improved in terms of increasing low RoB during 2000–2019.

Out of a total of 390 studies, 144 studies appeared in more than one Cochrane review, with inconsistent RoB assessments in 107 (74.3%) of them. The assessment of attrition bias (incomplete outcome data; *n* = 207, 53.1%) and selection bias (allocation concealment; *n* = 34, 8.7%) had the highest and lowest rates of disagreement between the different reviews, respectively.

### 3.3. The Overall Risk of Bias (ORoB) and Its Association with the Study Characteristics

After the removal of duplicate studies (which had not reached consistent assessments concerning the RoBs—determined in the Cochrane reviews), 2475 studies remained to calculate the ORoB. Almost half of these studies (*n* = 1119) had a high ORoB compared to 11.9% (*n* = 294) with a low ORoB.

The following journals had the highest percentage of studies with low ORoBs (considered eligible with at least ten included studies): *The European Journal of Oral Implantology* (73.1%, of 26 studies), *Journal of Endodontics* (52.3%, of 44 studies), and *Anesthesia Progress* (38.5%, of 13 studies). The following journals had the highest percentage of studies with high ORoBs (considered eligible with at least ten included studies): *Journal of Dentistry for Children* (91.7%, of 12 studies), *European Archives of Paediatric Dentistry* (81.8%, of 11 studies), and *Cancer* (74.2%, of 31 studies). Among the countries with more than 50 included studies, Italy and China had the highest percentage of low (32 out of 155, 20.6%) and high (49 out of 89, 55.1%) ORoBs, respectively.

Of the 821 studies conducted before the year 2000, 6.6% had low ORoBs, compared to 14.5% among those conducted after 2000 and 15.5% of those conducted after 2010. The studies that used placebos had a higher percentage of low ORoB (14.8% vs. 10.7%) and lower percentages of high ORoB (27.6% vs. 52.7%). The double- and triple-blind studies had higher percentages of low ORoB (23.6% and 23.3%, respectively), while the non-blinded studies had the lowest percentage of low ORoB (1.0%) and the highest percentage of high ORoB (80.4%). The studies with a crossover design had the highest percentage of low ORoB (28.8%), followed by cluster RCTs (11.9%), and parallel studies (11.2%). The quasi-experimental studies, predominantly, had a high ORoB (98.4%). The high-ORoB studies had the highest mean sample size (*n* = 414.3), followed by the unclear studies (*n* = 213.6), and those with low (*n* = 190.0) ORoBs (Table 1).

## 4. Discussion

Ranked 23rd out of 37 subjects in terms of number, the subject “dentistry and oral health” comprises 2.4% of all Cochrane reviews. This demonstrates a distinct shortage of evidence related to research in the field of dentistry, involving both interventional and diagnostic aspects compared to other medical disciplines [12]. Aside from the quantity, the quality of research is fundamental and needs to be continuously improved. For scoring the quality of dental clinical trials, RoB and other characteristics of the studies within Cochrane oral health systematic reviews were assessed.

Approximately half of the clinical trials (47.3%) showed a high ORoB compared to 11.1% with a low ORoB. This finding was consistent with the results reported by Yordanov et al., which revealed 43% of medical clinical trials with high ORoBs [13]. Earlier assessments of RoB in dental clinical trials showed a significant decrease in studies judged to have inadequate methodological standards using the Cochrane risk of bias tool [14]. On the other hand, no specific associations were identified to guide dental practitioners in optimising future clinical trials. The present findings are highly suggestive of the use of control groups in oral-health-related interventional studies, as they could noticeably reduce the ORoB. Furthermore, double- and triple-blind oral-health-related interventional studies showed lower ORoBs compared to unblinded studies. Quasi-experimental studies showed higher ORoBs compared to crossover, cluster RCT, and parallel designs. These findings may ultimately help clinical researchers to focus their studies within more organised settings and designs, and consider minimising probable RoBs in their relevant projects. Based on these findings, the use of crossover and parallel designs may be preferable for dental researchers. On the other hand, the high ORoB in split-mouth design studies (which is a widely used design among dental researchers) may require more detailed consideration by researchers when intending to use this type of RCT design, to determine if its use may not be suitable in some situations.

Although dentists are able to conduct high-quality studies with low risks of performance bias (95.9% of the double- and triple-blinded studies), apparently, they have not been eager to follow the blinding protocols of clinical interventional studies (cumulative percentage of double- and triple-blinded studies = 38.5%). This finding may be an implicit sign of a misunderstanding of dental research teams and the exclusive provisions of clinical research in dentistry. Predominantly, changing the exclusivity trend in research teams has been proved to impact the quality of evidence [15,16].

Despite knowing that the use of control groups decreases the ORoB, only 32% of the clinical trials reported having an arm as a control group. Additionally, among the various RoB domains, the performance bias (blinding of the participants and personnel) showed the highest rate of high risk (31.4% of the studies). Consequently, the development of both appropriate methodological strategies and a reporting protocol before conducting a clinical trial study is also imperative [13]. These goals can be ensured by seeking professional help from clinical methodologists and by consulting statisticians in planning studies and delineating their stages [15]. Providing better platforms for these communications, as well as constantly promoting their feasibility features, are critical [17]. Another long-term solution for decreasing the RoBs is to enhance the knowledge of dental students and dental practitioners about research methods [18]. Teaching research methods should be pursued in parallel with the provision of more feasible and convenient ways for dental clinicians to contact professional methodologists. In this connection, a recommended step is to consult a statistician in every study, as they can help refine the study design before the practical steps are taken. Patients are the ultimate stakeholders of these evidence-based decisions, given that they can be provided with the most reliable and up-to-date therapeutic options [19,20,21].

An interesting result of our study was that two journals in paediatric dentistry had the highest percentage of studies with a high ORoB. Conversely, the *Journal of Oral Implantology* and the *Journal of Endodontics*, two leading journals in their field, had more than 50% low ORoB, even with a considerable number of published articles included in Cochrane reviews. Paediatric dentistry researchers should bear in mind the need to improve the design of their studies, considering the fact that knowledge about the effectiveness of paediatric dentistry treatments has changed significantly [22,23,24]. Fortunately, this issue has recently been raised by researchers in paediatric dentistry [25].

This study takes into account the fact that the blinding of care providers is unachievable in some clinical trials in dentistry (e.g., surgical cases). Nonetheless, there are still various aspects of dental clinical trials that could potentially be improved by simple and feasible actions, such as the accurate reporting of sequence generation and allocation concealment or use of the most relevant study designs (as long as they are feasible).

One of the limitations of this study was the decision to only consider systematic reviews of interventional studies. This decision was made because systematic reviews of diagnostic and observational studies use different RoB assessment tools compared to interventional studies. This also helped us to better organize the findings and provide the results for most interventional reviews published by the Cochrane Oral Health group.

## 5. Conclusions

Almost half of the dentistry and oral-health-related clinical trials (47.3%) showed high ORoBs. Crossover and parallel designs and studies using a placebo as the control had a higher percentage of low ORoB compared to other designs. Therefore, according to the results of this study, dental research can benefit from robust designs to conduct studies with lower risk of biases.

## Figures and Tables

**Table 1:** ORoB (Overall Risk of Bias) for each study design and method.

	Low ORoB	High ORoB	Unclear ORoB
**Design**			
Cluster RCT	5 (11.9)	23 (54.8)	14 (33.3)
Crossover	44 (28.8)	39 (25.5)	70 (45.7)
Parallel RCT	219 (11.2)	863 (44.2)	871 (44.6)
Split-mouth	25 (9.9)	130 (51.4)	98 (38.7)
Quasi-experimental	0 (0)	62 (98.4)	1 (1.6)
Unclear	0 (0)	1 (100)	0 (0)
**Placebo**			
Yes	107 (14.8)	199 (27.6)	416 (57.6)
No	187 (10.7)	920 (52.7)	637 (36.6)
**Blinding**			
Single-blind	69 (12.3)	281 (50.1)	211 (37.6)
Double-blind	197 (23.5)	178 (21.2)	465 (55.3)
Triple-blind	14 (23.3)	22 (36.7)	24 (40.0)
Unblinded	4 (1.0)	336 (80.4)	78 (18.6)
Not reported	10 (1.8)	297 (52.6)	258 (45.6)

## Data Availability

Dataset is available in this figshare repository: https://doi.org/10.6084/m9.figshare.13070135 (accessed on 1 June 2021).
